# Suicidality and Its Relation with Physical and Mental Conditions: Results from a Cross-Sectional Study of the Nationwide Primary Care Population Sample in Latvia

**DOI:** 10.3390/medicina57090970

**Published:** 2021-09-16

**Authors:** Lubova Renemane, Anda Kivite-Urtane, Elmars Rancans

**Affiliations:** 1 Department of Psychiatry and Narcology, Riga Stradins University, Tvaika Street 2, LV-1005 Riga, Latvia; elmars.rancans@rsu.lv; 2 Department of Public Health and Epidemiology, Institute of Public Health, Riga Stradins University, Dzirciema Street 16, LV-1007 Riga, Latvia; anda.kivite-urtane@rsu.lv

**Keywords:** suicidality, mental disorder, physical condition, primary care

## Abstract

*Background and Objectives*: Physical and mental conditions are important risk factors for suicidality. However, there is no clear understanding of these relationships and the effect of co-occurrence on suicidality. We aimed to investigate the associations between current suicidality and mental disorders, physical conditions, and health-related factors in the nationwide primary care population in Latvia. *Materials and Methods*: A cross-sectional study was performed within the framework of the National Research Program BIOMEDICINE 2014–2017 at 24 primary care settings across Latvia in 2015. Adult patients were evaluated over one week at each facility. Socio-demographic variables, physical condition, and health-related factors were assessed on-site by trained psychiatrists. Mental disorders and suicidality were determined using the Mini International Neuropsychiatric Interview, and assessments were conducted over the telephone within two weeks after the visit to the general practitioner. *Results*: Of the 1485 cases, 18.6% reported suicidality. Only current depression, any anxiety disorder, any alcohol use disorder, and physical-mental multimorbidity were significantly associated with suicidality in the multivariate logistic regression analysis. Gastrointestinal diseases were associated with current depression alone (odds ratio (OR) 10.36; 95% confidence interval (CI) 2.34–45.76) and comorbid depression with any anxiety disorder (OR 7.55; 95% CI 2.15–26.49) among persons with current suicidality. *Conclusions*: Screening for depression, anxiety, and alcohol use disorders regularly among patients with physical illness may be important to help recognise suicidality in primary care that could improve the quality of life of patients and prevent suicides.

## 1. Introduction

Suicide is a complex medical worldwide phenomenon that leads to serious public health and social problems. According to the World Health Organization (WHO) report in 2018, approximately 800,000 people in the world die due to suicide each year, and suicide is the third leading cause of death among 15–19-year-olds in the general population [1]. It is also being recognized that suicide ideation and prior suicide attempts have been identified as important predictors of death by suicide [2,3]. Evidence indicates that for each death by suicide, 20 suicide attempts are estimated [4].

Suicidal ideation is highly prevalent in the general population (8.5%) [5] and primary care samples (12–32%) worldwide [6,7]. However, the first Latvian population-based study on self-reported suicidal behaviours in 2000 reported that the last year and lifetime prevalence of suicidal behaviours was 52.6 and 60.2%, and the incidence of suicide attempts was 1.8 and 5.1%, respectively [8]. Ten years later, the second Latvian population-based study demonstrated an 18.5% last year prevalence of suicidal behaviours and a 0.3% incidence of suicide attempts [9].

Physical and mental conditions are strongly linked and are estimated to be responsible for a large proportion of suicides [10,11,12]. The literature has consistently shown that most suicides are related to mental disorders, such as depression, substance use disorders, anxiety disorders, and schizophrenia, as the most relevant risk factors [10,13,14,15,16,17]. It is important to state that depression, anxiety, and substance use disorders are highly prevalent among primary care patients [18,19,20].

However, physical illness is rarely investigated as a motive for suicidality. Some studies have pointed out that several physical conditions are associated with an increased risk of suicide ideation and attempts. Black et al. reported that poor physical health, depression, psychosocial stress, interpersonal conflict, and panic increased the risk of suicidal ideation [14]. The epidemiological population-based study data from Australia suggest that thyroid disorders, syncope, seizures, liver disorders, and alcoholism are strongly associated with suicidal ideation [21]. A study from the United States highlighted that patients with multiple medical illnesses are at a high risk of suicidal behaviour [22]. Another retrospective study of persons who committed suicide from Germany reported that physical diseases were specified as a reason for committing suicide in 18.9% of cases, and mental disorders in 32.7% of cases. One third suffered from cancer and almost the same proportion of heart disease or chronic pain among persons with physical diseases. However, the authors conclude that patients with a long duration of both mental and physical disease are at a higher risk of committing suicide [11].

General practitioners (GP) play a crucial role in the early detection of patients with suicidal ideation and behaviour to provide appropriate care and prevent suicide [7,21,23]. The results of a population-based electronic case–control study of patterns of healthcare contacts in those who die by suicide suggest that help-seeking occurs in those at risk of suicide and escalates in the weeks before their death [24]. Moreover, the authors indicated that the last point of contact most often occurred in general practices and was most associated with mental health. Luoma et al. assumed that 45% of the people who complete suicide contact a primary care provider in the 30 days before their death [23].

Additionally, the WHO recognizes suicide as a public health priority and aims to lower the rate of suicide by 10% as part of a global target in the WHO Mental Health Action Plan 2013–2020 [25].

These facts highlight the requirement for detecting and evaluating risk factors of suicidality that helps in the early identification of people at risk, especially in the primary care population. Therefore, we aimed to investigate the associations between current suicidality and socio-demographics, mental disorders, physical conditions, and health-related factors in the nationwide primary care population sample in Latvia, as this information may predict patients at risk of suicide.

## 2. Materials and Methods

### 2.1. Study Design

A cross-sectional study was performed in 2015 within the framework of the National Research Program BIOMEDICINE 2014–2017 to assess the prevalence of mental disorders in Latvian primary care settings. The respondents were recruited from 24 primary care facilities (16 in urban and eight in rural areas) that covered all the health regions of Latvia. The following were the inclusion criteria: (1) treatment-seeking patients visiting a GP, (2) age ≥18 years, and (3) those who provided informed consent. Patients were excluded if they refused to participate in the study, were unable to participate because of their somatic condition (e.g., being deaf or mute), had an acute medical condition requiring urgent hospitalization, or were visiting their GP for administrative reasons. All participants were requested to answer a structured socio-demographic questionnaire and provide their medical measurements after signing an informed consent form before visiting their GP for one week at each primary care facility. Four trained psychiatrist interviewers conducted the Mini International Neuropsychiatric Interview (MINI), Version 6.0.0 over the phone within two weeks of the first contact with the patient [26]. The Riga Stradins University Ethics Committee approved this study and written informed consent was obtained from all participants.

### 2.2. Assessment

The MINI is a structured diagnostic interview for psychiatric disorders according to the Diagnostic and Statistical Manual of Mental Disorders, Fourth Edition and the International Classification of Disease 10th revision [26]. It is widely used for research purposes in psychiatric and general medical populations, including primary care patients. The MINI has been translated and adapted by authorship holders for use in 67 languages, including Latvian and Russian [27]. The interview was conducted over the telephone, which is acceptable and has been carried out in this way in other studies [28]. We used the MINI modules to identify current diagnoses of depressive episodes, recurrent depressive disorder, suicidality, alcohol use disorders (alcohol dependence or alcohol abuse), and anxiety disorders (panic disorders, agoraphobia, social phobia, obsessive compulsive disorder, posttraumatic stress disorder, or generalized anxiety disorder).

A structured interview in a GP office was carried out and all medical documentation available for the patients were assessed by trained psychiatrists. Questions used in structured interview to assess health behaviours and medical history were taken from the Health Behaviour Among Latvian Adult Population survey (previously FINBALT), bi-annually carried out by the Centre for Disease Prevention of Latvia since 1998 [29].

The structured socio-demographic questionnaire included questions about sex (male or female), age (18–34 years, 35–49 years, 50–64 years, or 65+ years), marital status (married or cohabiting, single, or live separately or divorced or widowed), employment status (employed, unemployed, or economically inactive), educational level (higher or unfinished higher education, general or vocational secondary or unfinished secondary education, or 9-year basic or unfinished basic education), and place of residence (urban: capital (Riga), another city, or rural).

The main medical reason for visiting the GP was determined by the question: “For what medical reason did you visit a GP today?” The responses were coded into eight categories: cardiovascular disease, diabetes, lung disease, gastrointestinal disease, arthritis, endocrine disease, oncological disease, and other reasons.

The respondents were asked about alcohol use and episodes of heavy drinking in the last year, “How often did you drink more than 1.5 L of beer, 600 mL of wine, 180 mL of vodka, or 5 standard drinks in the last 12 months?” The answers were categorized as: “every day or almost every day”, “3–4 times a week”, “1–2 times a week”, “1–3 times a month”, “less often”, or “never during the past year”.

Being overweight or obese was defined as having a body mass index (BMI) ≥25 kg/m^2^. BMI was calculated using the height and weight to the nearest 0.1 cm and 0.1 kg, respectively.

### 2.3. Participants

A total of 1756 patients who visited their GP were invited to participate in the study, and 152 refused to participate. The weighted response rate was 91.3% which ranged from 86.3–93.7% across 24 primary care settings in Latvia. Those who refused to participate did not differ significantly in terms of basic socio-demographic characteristics from the study sample. At baseline, 1604 patients were approached to complete the questionnaire, and 1585 participants had completed it. Among them, 100 patients who did not answer a follow-up telephone call thrice were excluded from the study. Therefore, the analysed sample comprised 1485 patients who were interviewed using the MINI over the telephone.

### 2.4. Statistical Analysis

Data were analysed using the Statistical Package for the Social Sciences (SPSS) version 20.0 (IBM, Armonk, NY, USA) [30]. Descriptive statistics (frequencies) were used to describe the study sample. Statistical significance of differences in the distribution of dependent variables between strata of independent variables were detected using the chi-square test or Fisher’s exact test. Factors associated with suicidality or medical reasons for GP visits were identified using binary logistic regression. Statistical significance was set at *p* < 0.05.

## 3. Results

### Characteristics of the Sample

Among the 1485 patients who approached a primary care GP, the current suicidality according to the MINI was identified in 18.6% (*n* = 276) of cases. Respondents with suicidality had a statistically higher rate of current depressive episodes (35.1%), recurrent depressive disorder (47.1%), anxiety disorders (33.3%), and alcohol dependence (6.5%) compared with the group without suicidality (4.5%, 10.7%, 11.8%, and 2.6%, respectively). Females (77.5%), people with general or vocational secondary education (53.5%), economically inactive individuals (51.3%), married individuals (50.2%), and those living in urban areas (52.5%) were more prevalent in the group with suicidality than in those without suicidality. There were no differences in relation to age, BMI, alcohol consumption, medical reason to visit the GP, alcohol use, and episodes of heavy drinking in the previous year between groups. The complete socio-demographic and medical characteristics of the study samples with or without current suicidality are listed in Table 1.

Table 2 represents the association of physical and mental conditions with current suicidality by multivariate logistic regression analysis adjusted for sex, age, level of education, employment status, marital status, and place of residence. Current mental disorders, such as depression (odds ratio (OR) 9.13; 95% confidence interval (CI) 3.01–27.68; Nagelkerke’s Pseudo R-Square 0.225), any anxiety disorders (OR 5.82; 95% CI 2.24–15.10; Nagelkerke’s Pseudo R-Square 0.140), any alcohol use disorders (OR 6.51; 95% CI 2.16–19.60; Nagelkerke’s Pseudo R-Square 0.093), and physical–mental multimorbidity (OR 10.80; 95% CI 1.91–61.03; Nagelkerke’s Pseudo R-Square 0.086) predicted concurrent suicidality. Neither multiple physical conditions nor separated groups (cardiovascular disease, diabetes, lung disease, gastrointestinal disease, arthritis, endocrine disease, oncological disease, administrative reasons, and other reasons) became evident as significant predictors.

Associations between physical and mental conditions among persons with current suicidality are shown in Table 3. Gastrointestinal diseases were associated with current depression alone (OR 10.36; 95% CI 2.34–45.76) and comorbidity with any anxiety disorder (OR 7.55; 95% CI 2.15–26.49) among persons with current suicidality. There was no association between depression, any anxiety disorder, any alcohol use disorder, depression and any alcohol use disorder, any anxiety and any alcohol use disorder, or a combination of all three mental disorders, including cardiovascular diseases, diabetes, lung diseases, arthritis, endocrine or oncological diseases, or other physical conditions.

## 4. Discussion

The present study highlights the suicidal risk factors and associations with medical conditions among patients who visited a primary care GP. Our results revealed that comorbid current mental disorders and physical-mental multimorbidity predicted concurrent suicidality in the multivariate logistic regression analysis when adjusted for socio-demographic variables. The most common comorbid current mental conditions associated with suicidality were depression, any alcohol use disorder, and any anxiety disorder. These findings are consistent with those of other studies. It is well known that mental disorders are important risk factors for suicide. One of the few studies carried out in primary care settings in Lithuania (the Baltic States) also found that the most important risk factors for suicidal ideation were current major depressive episodes, extensive alcohol consumption, and the use of antidepressants [31]. Another study conducted in primary care settings concluded that individuals with a greater burden of mental illness in terms of mood disorder comorbidity and depressive symptomatology are more likely to suffer from suicidal ideation [16]. Recent meta-analysis results show that mood disorders, substance use disorders, anxiety disorders, psychotic disorders, and personality disorders contribute to suicide in the general population [12]. Data from a large randomized controlled study in patients with anxiety disorders in primary care demonstrated that the presence of an anxiety disorder is associated with a greater frequency of suicidal thoughts and behaviours. The associated suicidal risk factors in this study were the presence of depression, mental health-related impairment, and lack of social support [32]. A study in an Arab primary care setting showed a high prevalence of depression, anxiety, and somatization among patients with physical illness. The most common mental conditions comorbid with physical illness were somatization or co-occurrence of somatization, depression, and anxiety. Additionally, significant associations were found for hypertension, asthma, and non-chronic medical conditions [33].

The data from primary care studies on the association between physical illness and suicidality are contradictory. Some studies have demonstrated an association between physical illness and suicidality, with another study denying this connection. A case–control study carried out in family practices in England suggested that coronary heart disease, stroke, chronic obstructive pulmonary disease, and osteoporosis were linked with elevated suicide risk. Moreover, the authors concluded that clinical depression is a strong confounder of increased suicide risk among physically ill people [34].

Our data demonstrated that current suicidality did not have any association with physical conditions alone but is associated with physical–mental multimorbidity. Our results are consistent with those of previous studies, which did not find a predictive role of physical illness in the risk of suicidality. A recent multiple cohort study following people for 20 years found that having only mental health conditions was associated with suicidal thoughts and suicidality, although it did not predict suicide-related items. Additionally, the authors suggested that mental and physical multimorbidity had a significant effect on suicidal thoughts and suicide attempts [35].

To highlight the physical-mental multimorbidity among persons with current suicidality, we found that current depression and co-occurrence of current depression and any anxiety disorders was associated with gastrointestinal diseases. Our findings are consistent with those of previous studies [11,36,37]. Additionally, the authors of a study of 1641 outpatients with gastrointestinal disorders concluded that most of the patients who seek medical consultation for gastrointestinal problems show associated affective and anxiety disorders [38]. Another study of 3256 depressed patients from the National Survey on Symptomatology of Depression reported that most subjects (70%) had concomitant gastrointestinal symptoms, and a higher frequency of gastrointestinal symptoms was associated with an increased risk of suicide ideation and attempts [39]. This may be conceptualized in the context of the psychosomatic relationship between physical and mental pathology and could be interpreted as overlapping symptomatology in gastrointestinal conditions, anxiety, and depression.

The reasons for the relationship between suicide and physical or mental morbidity remain unclear and requires additional future research. Our finding could be explained by the fact that patients who visit a family doctor regularly have more or less controlled physical illnesses and do not pose a risk of suicidality. However, mental illnesses are not fully controlled and are under-diagnosed in primary care settings; therefore, they are associated with suicidality. This statement supports the Latvian qualitative study in a primary care setting, suggesting that family doctors often do not recognize depression in patients presenting with somatic complaints [40]. Current knowledge about depression and management approaches of family physicians are insufficient; however, they acknowledge the importance and necessity of treating depression. This justifies the need for specific training programmes for GPs [41].

## 5. Limitations

There are several limitations to our study. The data demonstrated the prevalence of mental disorders and factors associated with these disorders only in a primary care population, which eliminates the potential to characterize individuals observed in specialized psychiatric outpatient departments, clinical settings, and the general population. Our target population involved persons visiting primary care settings; therefore, their health may differ from that of the general population. Another limitation is the clinical selection and sample size for certain diagnostic categories, such as arthritis and endocrine disease. Additionally, one of our research limitations could be a recall and response bias. We tried to decrease this bias by asking structured questions in the questionnaires by trained psychiatrists. The question categories have already been used in the Health Behaviour Among Latvian Adult Population survey (previously FINBALT), bi-annually carried out by the Centre for Disease Prevention of Latvia since 1998 [29]. Finally, due to the cross-sectional design of this study, it is impossible to conclude about the causality of the established links between common mental disorders and associated factors.

## 6. Conclusions

In the present cross-sectional study of the primary care population, we observed that current mental disorders, such as depression, any anxiety disorders, any alcohol use disorders, and physical–mental multimorbidity are risk factors for suicidality as measured by MINI. Considering this, family doctors need to pay attention to mental problems among patients with physical illnesses. They should be trained in screening patients for depression, anxiety, and alcohol use disorders to lower suicidality in the country. Additionally, it is important to visit a family doctor and control physical conditions on a regular bias because it could reduce suicidality. We suggest that the correct assessment and management of suicidality could improve the quality of life and prevent suicide. Thus, awareness of this relationship could help develop appropriate suicide reduction strategies and local suicide prevention action plans. Further research needs to be conducted to understand the complex of relationships between suicidality, physical conditions and mental illnesses in primary care patients.

## Figures and Tables

**Table 1:** Characteristics of study sample (*n* = 1485).

Variable ^a^ Values are Given as *n* (%) or Median (IQR)		Current Suicidality		
		Yes, *n* = 276; 18.6%	No, *n* = 1209; 81.4%	*p*
Age	18–34	42 (15.2)	169 (14.0)	0.74
	35–49	79 (28.6)	382 (31.6)	
	50–64	65 (23.6)	289 (23.9)	
	65+	90 (32.6)	367 (30.4)	
Sex	Male	62 (22.5)	391 (32.4)	**0.001**
	Female	214 (77.5)	816 (67.6)	
Education	Higher and unfinished higher education	72 (26.7)	370 (30.7)	**<0.001**
	General or vocational secondary and unfinished secondary	144 (53.3)	703 (58.3)	
	9-year basic, unfinished basic	54 (20.0)	132 (11.0)	
Employment status	Employed	113 (41.7)	673 (55.9)	**<0.001**
	Unemployed	19 (7.0)	65 (5.4)	
	Economically inactive	139 (51.3)	467 (38.8)	
Marital status	Married, cohabiting	136 (50.2)	770 (63.9)	**<0.001**
	Single	97 (35.8)	106 (8.8)	
	Live separately, divorced, widowed	38 (14.0)	329 (27.3)	
Place of residence	Capital (Riga)	61 (22.1)	248 (20.5)	**0.03**
	Other city	145 (52.5)	556 (46.1)	
	Rural	70 (25.4)	403 (33.4)	
Ethnicity	Latvian	169 (62.6)	750 (62.2)	0.85
	Russian	82 (30.4)	380 (31.5)	
	Other	19 (7.0)	75 (6.2)	
Diagnosis	Current any mental disorders	271 (100.0)	275 (23.0)	**<0.001**
	Current Depressive episode	97 (35.1)	54 (4.5)	**<0.001**
	Current Recurrent depressive disorder	130 (47.1)	129 (10.7)	**<0.001**
	Current anxiety disorders	92 (33.3)	143 (11.8)	**<0.001**
	Current any alcohol use disorder	25 (9.1)	45 (3.7)	**<0.001**
	Current Alcohol dependence	18 (6.5)	31 (2.6)	**0.02**
	Current Alcohol abuse	7 (2.5)	14 (1.2)	0.09
Alcohol use, episodes of heavy drinking in last year (5 or more doses of alcohol at once)	Every day/nearly every day	0 (0)	1 (0.1)	0.25
	3–4 times a week	1 (0.4)	2 (0.2)	
	1–2 times a week	3 (1.1)	14 (1.2)	
	1–3 times a month	12 (4.4)	42 (3.5)	
	Less often	20 (7.4)	141 (11.7)	
	Never during the past year	235 (86.7)	1006 (83.4)	
Body mass index (kg/m^2^)		26.8 (23.2–32.1)	27.8 (24.4–32.0)	0.52
Medical reason to GP’s visit	Cardiovascular disease	102 (37.6)	426 (35.3)	0.48
	Diabetes	15 (5.5)	50 (4.1)	0.33
	Lung disease	13 (4.8)	43 (3.6)	0.38
	Gastrointestinal disease	14 (5.2)	51 (4.2)	0.51
	Arthritis	7 (2.6)	40 (3.3)	0.53
	Endocrine disease	5 (1.8)	28 (2.3)	0.67
	Oncology	24 (8.9)	103 (8.5)	0.91
	Other ^b^	224 (82.7)	992 (82.3)	0.88

Statistically significant results are presented in bold font. The medical reason for a GP visit for one person could be a combination of different diseases. ^a^ The sum of stratified numbers may differ across variables due to missing values. ^b^ Kidney, liver, infectious diseases, seasonal infections. IQR: the interquartile range.

**Table 2:** Association of physical and mental conditions with current suicidality in multivariate logistic regression analysis.

Variable	Current Suicidality		
	OR	95% Cl	*p*
Any current mental disorders	3.16	0.19–52.58	0.42
Current depression ^1^	**9.13**	**3.01–27.68**	**<0.001**
Any current anxiety disorders	**5.82**	**2.24–15.10**	**<0.001**
Any current alcohol use disorders	**6.51**	**2.16–19.60**	**0.001**
Physical conditions only ^2^	1.03	0.68–1.55	0.89
Multimorbidity ^3^	**10.80**	**1.91–61.03**	**0.007**
Neither physical nor mental condition	1		

Adjusted for sociodemographic characteristics: sex, age, level of education, employment status, marital status, place of residence. Statistically significant results are presented in bold font. ^1^ Depressive episode, recurrent depressive disorder—current depression from MINI. ^2^ Medical reason for GP visit. The analysis of separate physical conditions: cardiovascular disease, diabetes, lung disease, gastrointestinal disease, arthritis, endocrine disease, oncology, other, administrative reason did not show statistically significant results. ^3^ One or more mental and one or more physical illness in the same person.

**Table 3:** Association of physical and mental conditions among persons with current suicidality in multivariate logistic regression analysis.

	Medical Reason for General Partitions’ Visit							
Current Mental Disorders	Cardiovascular Disease	Diabetes	Lung Disease	Gastrointestinal Disease	Arthritis	Endocrine Disease	Oncology	Other
Depression ^2^	0.68	0.77	0.82	**0.002 ^3^**	0.33	0.96	0.77	0.21
Any anxiety	0.40	0.06	0.14	0.20	0.86	0.71	0.50	0.49
Any alcohol used	0.66	0.99	0.79	- ^1^	0.99	0.99	0.35	0.72
Depression and any anxiety	0.62	0.24	0.09	**0.002 ^4^**	0.81	0.99	0.92	0.13

Adjusted for socio-demographic characteristics: sex, age, level of education, employment status, marital status, place of residence. Statistically significant results are presented in bold font, *p* < 0.05. ^1^ There are no cases. ^2^ Depressive episode, recurrent depressive disorder—current depression from MINI. ^3^ OR 10.36; 95% CI 2.34–45.76; Nagelkerke’s Pseudo R-Square 0.309; ^4^ OR = 7.55; 95% CI 2.15–26.49; Nagelkerke’s Pseudo R-Square 0.280. Comorbid “Depression and any alcohol used disorders”, “Any anxiety and any alcohol used disorders”, and “All three mental disorders” did not show statistically significant associations with physical conditions or lacked any cases in crosstabulations.

## Data Availability

The data presented in this study are available on request from the corresponding author. The data are not publicly available because they involve sensitive information.
